# Gadamerian philosophical hermeneutics as a useful methodological framework for the Delphi technique

**DOI:** 10.3402/qhw.v10.26291

**Published:** 2015-05-05

**Authors:** Diana Guzys, Virginia Dickson-Swift, Amanda Kenny, Guinever Threlkeld

**Affiliations:** 1 La Trobe Rural Health School, La Trobe University, Bendigo, Australia; 2 La Trobe Rural Health School, La Trobe University, Albury-Wodonga, Australia

**Keywords:** Health and well-being methods, dialogue, collective opinion, democratic group method

## Abstract

In this article we aim to demonstrate how Gadamerian philosophical hermeneutics may provide a sound methodological framework for researchers using the Delphi Technique (Delphi) in studies exploring health and well-being. Reporting of the use of Delphi in health and well-being research is increasing, but less attention has been given to covering its methodological underpinnings. In Delphi, a structured anonymous conversation between participants is facilitated, via an iterative survey process. Participants are specifically selected for their knowledge and experience with the topic of interest. The purpose of structuring conversation in this manner is to cultivate collective opinion and highlight areas of disagreement, using a process that minimizes the influence of group dynamics. The underlying premise is that the opinion of a collective is more useful than that of an individual. In designing our study into health literacy, Delphi aligned well with our research focus and would enable us to capture collective views. However, we were interested in the methodology that would inform our study. As researchers, we believe that methodology provides the framework and principles for a study and is integral to research integrity. In assessing the suitability of Delphi for our research purpose, we found little information about underpinning methodology. The absence of a universally recognized or consistent methodology associated with Delphi was highlighted through a scoping review we undertook to assist us in our methodological thinking. This led us to consider alternative methodologies, which might be congruent with the key principles of Delphi. We identified Gadamerian philosophical hermeneutics as a methodology that could provide a supportive framework and principles. We suggest that this methodology may be useful in health and well-being studies utilizing the Delphi method.

Researchers interested in harnessing expert knowledge on a broad range of health and well-being topics are increasingly using Delphi as a means of capturing collective opinion. The method has been adopted across a wide range of health, social care, and well-being studies related to policy, clinical practice, planning, and evaluation. Whilst the use of Delphi is increasing in health and well-being research, less attention has been given to the methodological underpinnings of the Delphi method. Our aim in this paper is to present Gadamerian philosophical hermeneutics as a methodological framework that others may find useful when undertaking health and well-being research using the Delphi method.

We were interested in the Delphi method (Delphi) for its usefulness for addressing research questions where diverse perspectives exist or knowledge is incomplete. Our specific interest was fuelled by our involvement in a large community-based health and well-being study. We explored Delphi as a method for facilitating anonymous conversations between participants about health literacy, as a mechanism for capturing collective views (Keeney, McKenna, & Hasson, 2011; Linstone & Turoff, 2011). However, consistent with the views of Gorman (2011), we believed that an understanding of the methodology that underpinned Delphi was fundamental in providing the framework and principles for our work, and central to research integrity. Researchers argue that there is often confusion between the terms methodology and method; however, we are clear that methodology refers to the rules, strategy, design principles, or frame of reference, which are influenced by the paradigm that guides the research method undertaken (McGregor & Murnane, 2010; Tracy, 2012).

We completed an initial literature search to identify the underpinning methodology of Delphi but found little guidance. We postulate that this results from the manner and era in which Delphi originated. Delphi developed in a period when the scientific method of research was dominant and research was outcome driven, rather than emerging from a philosophical position. Helmer (1967), a member of the RAND Corporation credited with developing Delphi, described Delphi as a systematic method to obtain the relevant intuitive insight and judgement of experts in the absence of a proper theoretical foundation. Contemporary researchers in the health and social sciences are more aware of the pivotal role methodology plays in inductive research. Therefore, we sought to map recent thinking related to methodologies informing use of Delphi.

Using the scoping review framework of Arksey and O'Malley (2005), we sought to identify health and social science articles published between 2010 and 2014 which report Delphi as the research method. The results of the scoping activity are presented in this article to illustrate our issue of concern. A majority of researchers who select Delphi as their research method do not report, or perhaps may not consider, the methodological underpinnings of their work. This may reflect a pervasive indifference to methodology, or avoidance resulting from epistemological confusion. Admittedly, it may simply result from the lack of space provided for such discussion in journals. Yet, this omission is of little value for those who seek theoretical foundations.

Seeking to identify a philosophy that provides a methodological rationale retrospectively is problematic, yet to do so may increase the rigour and value of studies using Delphi. The scoping review assisted in identifying the views of other researchers who had similarly struggled with this problem. Our review provided a platform from which we were able to consider alternative methodologies that might be congruent with the key principles of Delphi. The cyclical process of engaging with text and reflection exemplified through hermeneutics resembled the process which is fundamental to Delphi. Another core aspect of the method is that the process seeks to challenge personal perspectives, reshaping these through consideration of the perspectives of others. Despite Gadamerian hermeneutics developing from interpretation of historical and most frequently religious texts, we believe that the philosophy of thought, or ontological process Gadamer theorizes, provides understanding of the iterative interpretive process that occurs when using Delphi. We therefore propose that Gadamerian philosophical hermeneutics could provide a suitable methodological framework for health and well-being researchers using the Delphi method. We present our argument for this, in the belief that this may be useful for others to consider.

## Background

### The Delphi technique

Delphi assists in pooling expert knowledge to develop a collective opinion on a specific topic. It was developed to facilitate group communication for structural modelling of weapon requirements for the military following World War II (Donohoe & Needham, 2009; Linstone & Turoff, 2011). Due to national security concerns, publication relating to early Delphi did not occur until 10 years after they took place within the RAND Corporation (Dalkey & Helmer, 1963). Helmer (1967) suggest that Delphi developed in response to the rapid change caused by advances in technology, resulting in an increased recognition for the need to plan for future possibilities or forecasting, rather than simply being reactive. Delphi was considered a systematic approach to explore the factors that influence individual judgement, and bring factors which participants may not have considered through provision of a summary of others views (Dalkey & Helmer, 1963). It was presented as an experiment, where repeated intensive questioning of individual experts occured to ensure direct confrontation between experts was avoided. Questions were designed to make the reasoning behind the participant's perspective apparent, and how information from unknown others influenced original perspectives.

The central premise of Delphi is the generation of knowledge that results from dialogue achieved via organized interaction between knowledgeable individuals (Fletcher & Marchildon, 2014). Idea generation or brainstorming occurs through open-ended questions posed in an initial survey round (Skulmoski, Hartman, & Krahn, 2007). Traditionally, in subsequent rounds, participants are provided with a summary of the previous survey responses to consider. Participants review this, rank or indicate their level of agreement to the responses of others, and provide the reasoning for their opinion (Knott et al., 2012). Multiple survey iterations promote insightful decision-making through the anonymous re-evaluation by participants of their own view in the light of other opinions (Paraskevas & Saunders, 2012). Additional survey rounds continue until stability of responses occurs, which reflects achievement of theoretical saturation, although in some studies survey rounds cease once a predetermined level of consensus is achieved (Mamaqi, Miguel, & Olave, 2011; Paraskevas & Saunders, 2012). Stability of opinion is achieved when each participant has had the opportunity to consider and understand the views of others, but there are no further shifts in responses, signalling consensus and illuminating areas of difference.

Pooling of expert knowledge is one of the strengths of Delphi over research methods that focus on individual opinion, such as interviews (Snape et al., 2014). Delphi has been described as combining the collaborative effect of focus groups with the rigour of traditional surveys (McIntyre, Novak, & Cusick, 2010). Identifying the specific differences in Delphi from other group research methods assists in highlighting key methodological principles. In Delphi, participants do not interact directly with others but rely on text to share their opinions, responding to the research question in writing. The responses are collated and participants are then informed about others’ responses. Following reflection on the responses of others, participants are invited to adjust their response should they wish to. This process is repeated until stability of opinion is reached. The nominal group technique (NGT) is similar to Delphi, however, there is direct interaction between NGT group members to discuss or clarify ideas, removing the advantage of anonymous interaction provided by Delphi (Binnendijk, Gautham, Koren, & Dror, 2012). The four key components that define a process as the Delphi method are anonymity, iteration, controlled feedback, and aggregation of group response (Skulmoski et al., 2007). Anonymity is maintained throughout Delphi by reliance on written responses.

The advantage of using the Delphi method over other group research methods, such as focus groups and nominal groups, is in minimizing the influence of group dynamics on the findings (Fletcher & Marchildon, 2014). Face to face interactions have the potential for some participants to modify their responses in deference to the opinions expressed by other participants, particularly those who they may perceive as their superiors. A participant with a dominant personality may inhibit others within a group from expressing a different point of view. These possibilities are avoided through anonymous interaction, resulting in a more democratic process in which all perspectives are equally included. A further advantage of the method is that it facilitates the inclusion of geographically dispersed participants, as the process does not require participants to congregate in a single location. The time involved in undertaking the traditional Delphi method is sometimes considered its weakness.

A number of modifications to Delphi, including omission of the primary exploratory step in the process, have occurred over time. The Ranking Delphi, does not include an initial open-ended question survey round, but rather provides content often derived from the literature or other sources. A Delphi-like process is then used to rank or prioritize, using multiple survey rounds with the aim of reaching consensus, with regard to the order or level of agreement with the provided content. The “real time Delphi,” where the multiple survey rounds are compressed into a single meeting is another modification to the classical Delphi method. This potentially reduces the time available for thoughtful reflection of one's own perceptions in light of other responses. Consideration of disagreement is valuable, as stable disagreement is recognized as being informative, highlighting differences in perspectives (Goluchowicz & Blind, 2011; Linstone & Turoff, 2011; Rowe, Wright, & McColl, 2005). The value of stable disagreement is particularly valued in what is referred to as the “policy Delphi,” where development of various options is used to inform decision-making.

### The use of Delphi in health and well-being research

The use of Delphi in studies that explore questions related to health and well-being is increasing. In health and well-being policy, recent examples include the creation of activity-friendly environments for children (Aarts, Schuit, Van De Goor, & Van Oers, 2011) and access to wireless technologies for people with disabilities (Baker & Moon, 2010). Delphi has been used in studies seeking to develop practice guidelines and competencies in the area of health and well-being. The development of definitions of best practice in child protection (Ager, Stark, Akesson, & Boothby, 2010), peer support guidelines in high-risk organizations (Creamer et al., 2012), and guidelines for caregivers of people with bipolar disorder (Berk, Jorm, Kelly, Dodd, & Berk, 2011) are examples of this application. Clinical healthcare practice has been advanced through Delphi studies, including the development of practice guidelines for the management of head injuries (Undén, Ingebrigtsen, & Romner, 2013), professional competencies for optometrists (Myint, Edgar, Kotecha, Crabb, & Lawrenson, 2010), and identifying clinical indicators for musculoskeletal ultrasound (Klauser et al., 2012).

Delphi has proved a valuable research method in the development of resources and tools for diverse healthcare needs including a stammering information programme (Berquez, Cook, Millard, & Jarvis, 2011); the suicidal patient observation chart (Björkdahl, Nyberg, Runeson, & Omérov, 2011); and the domains of quality of life (Pietersma, De Vries, & Van den Akker-Van Marle, 2014). Researchers focused on planning and evaluation in health and well-being often report using Delphi. Examples include the examination of elements of a new model of adaptive adult bereavement (Doughty, 2009) and planning education to address the needs of first responders regarding survivors’ psychosocial reactions (Drury et al., 2013). Identifying a suitable methodological underpinning for Delphi should assist in ensuring that the increasing volume of health and well-being studies using this method are well designed, and researchers rigorous in their approach.

### The scoping review

Recognizing that Delphi was not a research method historically aligned to a specific methodology, we sought to understand what methodology contemporary researchers identified as a suitable supportive framework. We utilized a scoping review method to provide a rigorous and transparent approach to mapping relevant literature that would provide insight into the methodologies underpinning recent Delphi studies. Arksey and O'Malley (2005) recommend a multistage approach for undertaking scoping reviews. Consistent with their approach, in the first stage we developed our research question, “what methodological explanation is provided in contemporary research studies for selecting Delphi as a research method?” and broad key terms to capture the broadest pool of data (Arksey & O'Malley, 2005). Drawing on a wide range of health and social science research literature, a search was undertaken in September 2014 using ProQuest, CINAHL, Expanded Academic, and Scopus databases for the second stage in the scoping process.

The practicalities of time and cost limitations required us to establish clear criteria (Hammersley, 2011). Included articles were required to be published in English between 2010 and 2014 in peer-reviewed health and social science journals. The initial search sought articles that made reference to the “Delphi technique,” “Delphi method,” or “Delphi approach” in the abstract. This initial search resulted in 3056 articles. Use of the terms theoretical perspective, philosophy, or method* anywhere within the articles was then included to further refine this search. The use of a Boolean signifier (*), with a truncated word, enabled the search to include all terms from the same root. Therefore articles that discussed methodology would be included in the data pool by using method* as the second search term. The result of this two-stage data base search is detailed in Table I.

**Table I:** Results of data base search.

Data base	ProQuest	CINAHL	Expanded academic	Scopus	Total articles
Delphi technique/Delphi method/Delphi approach (in abstract)	680	48	11	2317	3056
Methodology/philosophy/theoretical perspective (in text)	163	3	8	192	366

**Figure 1:**
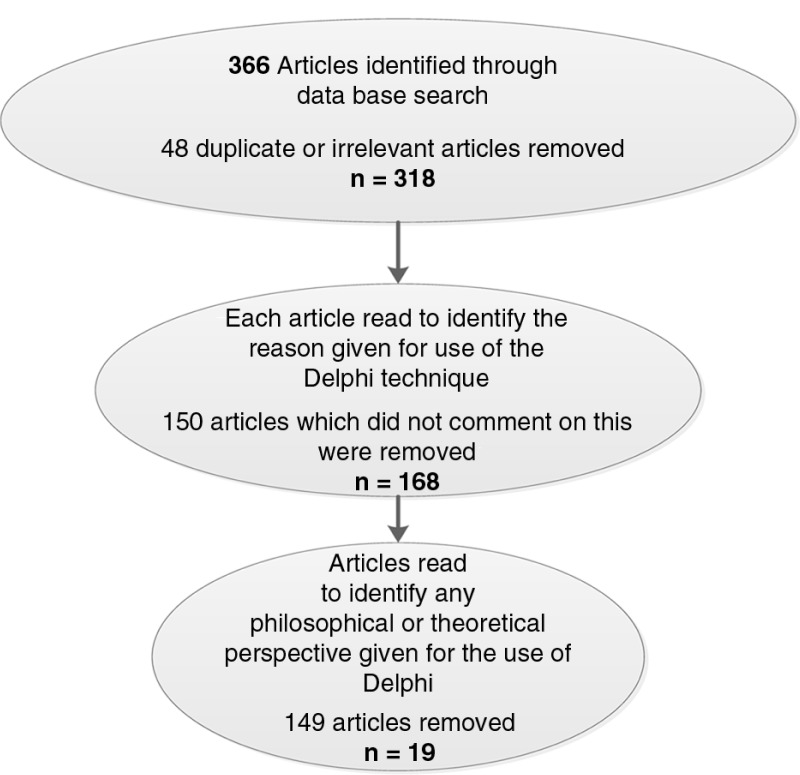
Summary of the review process.

The refined database search resulted in a more manageable 380 articles, from which 42 duplicate articles were removed. The remaining 338 articles were then read individually to determine what rationale was given by the authors for using Delphi in research. During this stage, four articles were found to discuss Delphi as a location in Greece or ancient Greece, and two articles discussed Delphi in terms of mathematical language. As these articles did not contain information relevant to our review, they were removed from the data pool. A further 150 articles were removed as the authors did not provide any rationale for the use of Delphi. Of the remaining 149 articles, the stated justification for use of the Delphi research method related to specific aspects of the processes, predominantly the desire to achieve consensus or convergence of opinion. Several authors qualified this, saying that use of Delphi resulted specifically in the most “reliable” consensus (Schmiedel, vom Brocke, & Recker, 2013; Vakani & Sheerani, 2012; Xia & Chan, 2012). However, other researchers emphasized the benefit of identifying areas of disagreement through the use of Delphi (Snape et al., 2014; Tuominen, Tapio, Varho, Järvi, & Banister, 2014; Warth, von der Gracht, & Darkow, 2013). Other rationales provided can be broadly categorized as highlighting collaboration (Ferguson, Ireland, & Ireland, 2013; Munguatosha, Muyinda, & Lubega 2011; Nworie, 2011); structured group communication (Dikmen, Birgonul, Ozorhon, & Sapci, 2010; Keyvanfar et al., 2014; Manley & Zinser, 2012); the value of iteration and reflexivity (Ifinedo & Ifinedo, 2011; Loblaw et al., 2012; Venhorst, Zelle, Tromp, & Lauer, 2014); and greater democracy through anonymity (Chen, Wakeland, & Yu, 2012; O'Rourke et al., 2014; Venhorst et al., 2014). Only 19 articles were judged to provide some reference to a methodology, philosophy, or theoretical perspective. The PRISMA framework that guided this process is represented in Figure 1.

The final three stages of the scoping review process involved collating or charting the data, summarizing, and reporting results. Consistent with the third stage of Arksey and O'Malley scoping review framework, the author, publication year, article title, journal, research aim, and methodological statements are charted in Table II.

**Table II:** Charting the scoping review results.

Author	Year	Title	Journal	Aim	Methodological comments
Brody et al.	2012	Identifying components of advanced-level clinical nutrition practice: A Delphi study	Journal of The Academy of Nutrition and Dietetics, 112(6), 859–869	The purpose of this research was to gain expert consensus on the essential characteristics and activities that define an advanced practice registered dietitian who provides clinical nutrition care to patients or clients	Delphi is described as “deriving quantitative estimates through qualitative approaches”
Chan, Wey, & Chang	2014	Establishing disaster resilience indicators for Tan-sui river basin in Taiwan	Soc Indic Res 115, 387–418	This paper proposes an application that combines fuzzy Delphi and analytic network process techniques in order to establish a set of disaster resilience indicators for a re-developed urban area in Tan-sui River Basin (Taiwan)	Delphi is referred to as a reliable qualitative research method
Diam, Laakso, Rubin, & Linturi	2012	The role of regulation in the mobile operator business in Finland	Foresight, 14(2), 154–67	In this study, the authors used the Delphi method for estimating the causes and effects of laws and other regulations impacting on mobile operator business in the past few decades, and consider potential effects in the years 2010–2015	The authors state that Delphi research may be catagorized as either quantitative or qualitative study
Hamilton, Coldwell-Neilson, & Craig	2014	Development of an information management knowledge transfer framework for evidence-based occupational therapy	VINE: The journal of information and knowledge management systems, 44(1), 59–93	The purpose of this paper is to present an information management knowledge transfer (IM-KT) framework which emerged from a study looking at digital literacy in the occupational therapy profession. Phase 3 of this study used the Delphi method to explore how occupational therapy could advance as a digitally literate profession refining the conceptual framework developed over phases 1 and 2	Phase 3 of this research is described as using a *Kantian* or contributory Delphi approach
Hanekom et al.	2012	Reaching consensus on the physiotherapeutic management of patients following upper abdominal surgery: A pragmatic approach to interpret equivocal evidence	BMC Medical Informatics and Decision Making, 12, 5	The aim of this paper is to develop evidence-based clinical management algorithm for the management of patients following abdominal surgery through a Delphi process of consensus	The Delphi is identified as a *pragmatic* methodology
LaBelle	2012	Constructing post-carbon institutions: Assessing European Union carbon reduction efforts through an institutional risk governance approach	Energy Policy 40, 390–403	This paper examines three different governance approaches the European Union and member states are relying on to reach a low carbon economy by 2050	The authors of this Delphi study state that it relies on a qualitative *grounded research approach*
López-Sánchez, & Pulido-Fernández	2014	Incorporating sustainability into tourism policy: A strategic agenda for Spain	European Journal of Tourism Research, 7, 57–78	This paper proposes a methodology for incorporating sustainability into tourism policy. Delphi analysis was initiated, with the aim of obtaining an assessment of the major errors and problems of the current Spanish tourism policy on sustainability	Delphi is explained to be a forecasting technique for obtaining qualitative or subjective information, which are quantified statistically, through measures such as; mean, median, and quartiles
Mason & Nair	2013	Supply side strategic flexibility capabilities in container liner shipping	The International Journal of Logistics Management, 24(1), 22–48	The purpose of this paper is to explore the extent to which supply side flexibility tactics are deployed by operators in the container liner shipping sector in 2009/2010 to restrict supply in a market which is characterized by over-supply (as well as under demand)	The authors describe their study as a mixed method approach, where knowledge is supplemented throughout by a qualitative Delphi-based research methodology
McNichols	2010	Optimal knowledge transfer methods: A Generation X perspective	Journal of Knowledge Management, 14(1), 24–37	This research study seeks so explore the thoughts and perspectives of Generation X aerospace engineers regarding strategies, processes, and methods to enhance the transfer of knowledge from Baby Boomers to Generation X aerospace engineers	Delphi is described as a qualitative research method
Meng, Xiuwei, & Anli	2011	A theoretical framework of caring in the Chinese context: A grounded theory study	Journal of Advanced Nursing, 67(7), 1523–1536	This paper reports a study that describes the components of nurse caring in the Chinese cultural context	The authors state that a *grounded theory research* design using the Delphi method were adopted in this study
Palo & Tähtinen	2011	A network perspective on business models for emerging technology-based services	Journal of Business & Industrial Marketing, 26(5), 377–388	This study seeks to identify the generic elements of a business model in the field of technology-based services and uses those elements to build a networked business model	The authors describe this as a qualitative futures study employing the Delphi method, which empirically grounds the concept with a variety of views, perceptions, and opinions
Paraskevas & Saunders	2012	Beyond consensus: An alternative use of Delphi enquiry in hospitality research	International Journal of Contemporary Hospitality Management, 24(6), 907–924	In this paper the authors reflect on the research methodology of a project that explored organizational crisis signals detection using Policy Delphi with a criterion sample comprising 16 senior hotel executives involved in crisis management	Delphi is presented as *phenomenological research* which relies on the elicitation and subsequent analysis of expert-participant opinion from individuals who were part of the phenomenon under study and had their own unique experiences and deep understandings of the issues of concern
Pérula et al.	2012	Is the scale for measuring motivational interviewing skills a valid and reliable instrument for measuring the primary care professionals motivational skills?: EVEM study protocol	BMC Family Practice, 13, 112	The researchers in this project try to test the hypothesis that a tool called “Assessment Scale motivational interviewing” (EVEM in Spanish) designed to assess whether the Spanish doctors have MI skills to promote in their patients behavioural changes have good psychometric properties, in terms of validity and reliability	The authors describe Delphi as a qualitative study obtaining expert opinions
Santos & Gomes	2010	Operating room information systems adoption by Portuguese clinical users	WSEAS Transactions on Communications, 10(9), 626–635	This research aims to assess the impact of the adoption of information systems by clinical users in the operating room	The Delphi method is presented as a method of qualitative research that aims to obtain a qualified opinion about certain issues, from a group of selected individuals
Sobaih, Ritchie, & Jones	2012	Consulting the oracle? Applications of modified Delphi technique to qualitative research in the hospitality industry	International Journal of Contemporary Hospitality Management, 24(6), 886–906	This paper aims to discuss the classical Delphi and its advantages and disadvantages in qualitative research, particularly in hospitality	The authors state that the articulation, interpretation and testing of the experts’ belief systems is the basis of the *interpretivist research paradigm* underpinning the Delphi technique
Tang & Wu	2010	Obtaining a picture of undergraduate education quality: A voice from inside the university	Higher Education, 60(3), 269–286	This study aims to construct ranking indicators from the perspective inside of the university and shift the ranking target from overall university quality to undergraduate education quality	The authors state that Delphi is a methodology by which subjective data can be transformed into quasi-objective quantitative data and to facilitate decision-making of controversial issues
Traynor, Boyle, & Janke	2013	Guiding principles for student leadership development in the doctor of pharmacy program to assist administrators and faculty members in implementing or refining curricula	American Journal of Pharmaceutical Education, 77(10), 1–10	To assist administrators and faculty members in colleges and schools of pharmacy by gathering expert opinion to frame, direct, and support investments in student leadership development	The Delphi is described as a qualitative research technique that requests and refines the collective thoughts and opinions of a panel of experts
Van Kemenade, Hardjono, & De Vries	2011	The willingness of professionals to contribute to their organization's certification	International Journal of Quality & Reliability Management, 28(1), 27–42	This paper seeks to find out which factors influence the willingness of professionals to contribute to a certification process and to understand the rationale behind this willingness	The Delphi is described as a qualitative research technique
Wilson	2011	New-school brand creation and creativity—Lessons from Hip Hop and the global branded generation	Journal of Brand Management, 19(2), 91–111	The stated aim of this paper is to report an Expert Delphi study which aims to present a new hip hop inspired model for brand creation; and second to offer an innovative approach to in depth qualitative studies, using “word cloud” software	Delphi offers a method by which a consensus of understanding can be reached in a wider context using *a cyclical hermeneutical approach* to qualitative opinion-based feedback

Interpretivism, focused on understanding the meaning of the research topic from the perspective of the participants, was the overarching epistemology given in most of the 19 articles that described some methodological principle in relation to Delphi. Reference was simply made to a qualitative research approach in six of the studies (Chan, Wey, & Chang, 2014; Daim, Laakso, Rubin, & Linturi, 2012; McNichols, 2010; Pérula et al., 2012; Santos & Gomes, 2010; Traynor, Boyle, & Janke, 2013; Van Kemenade, Hardjono, & De Vries, 2011), which is generally consistent with interpretivist epistemology and constructivist ontology (Andrews, Sullivan, & Minichello, 2004). A qualitative approach to achieve quasi-objective quantitative estimates was discussed in four studies (Brody, Byham-Gray, Touger-Decker, Passannante, & Maillet, 2012; López-Sánchez & Pulido-Fernández, 2014; Palo & Tähtinen, 2011; Tang & Wu, 2010). However, Hanekom et al. (2012) simply described Delphi as a pragmatic methodology, without further explanation. Sobaih, Ritchie, and Jones (2012) clearly stated that the Delphi study they undertook sat within the interpretivist research paradigm. They presented case studies illustrating Delphi research, stating that these cases highlight that Delphi has a “multi-paradigmatic” but consensual nature.

The authors of only five studies identified what could be considered philosophical perspectives proposed as underpinning Delphi research. LaBelle (2012), as well as Meng, Xiuwei, and Anli (2011), have used the research data generated through Delphi to generate theory. In grounded theory an iterative approach to data analysis is adopted which resonates with the iterative process of the Delphi surveys rounds that seek to clarify and consolidate the data. Paraskevas and Saunders (2012) do not provide any explanation for labelling Delphi phenomenological research, other than to say that researchers construct knowledge through the collection of multiple sets of interpretations, involving participants in the data co-creation and interpretation of the phenomenon being studied. Browne (2004), however, explains how phenomenology differs from grounded theory, as the intention is to describe the phenomena, rather than develop a theory from it. Hamilton, Coldwell-Neilson, and Craig (2014) refer to a Kantian or contributory Delphi approach, with no further explanation. Wilson (2011) incorporates Delphi in what he describes as a cyclical hermeneutical approach. Hermeneutics focuses on the interpretation of meaning (Andrews, Sullivan, & Minichello, 2004), particularly how we come to understand the meaning of text or art (Gadamer, 1996). The emphasis on a cyclical process reinforces the significance of the iterative process of the Delphi method.

## Discussion

A number of variations of Delphi have developed since its inception. Critics of the Delphi method have sometimes described these as inconsistencies in the Delphi process, which may possibly result from the absence of a universally recognized methodology to guide research practice. Mitroff and Turoff (2002) argue that there is no single or best philosophical basis that underpins Delphi. However, the stance adopted in research is more than simply philosophical interest, as it influences application, and therefore results. The absence of an appropriate philosophical foundation will result in inconsistent conceptualizations, with potential for poor research practice and less convincing results (Gorman, 2011; McGregor & Murnane, 2010). The philosophical awareness of researchers strengthens the intellectual consistency and rigor of research processes and the value of findings. Reflection on the aim of the research activity is necessary to select and justify the approach adopted in any study. Attention to philosophical issues is particularly critical when several competing approaches are possible, and choice of approach should be guided by scrutiny of the underlying philosophical assumptions (Hammersley, 2011).

Retrospective identification of a philosophical perspective that is congruent with the Delphi method is admittedly problematic, and may well be contested. As Delphi did not develop from a specific philosophical view, it is unlikely that any single philosophy will seamlessly support this research method. Identifying a philosophical framework that provides a good fit and logical support for Delphi may assist in maintaining research integrity. Several philosophies have been suggested in association with Delphi to provide a foundation which meets each researcher's unique needs, including those represented by Locke, Leibniz, Kant, Hegel, and Singer (Mitroff & Turoff, 2002). Yet the results of our scoping review suggest that these philosophies do not meet the need of many researchers, as illustrated by the paucity of researchers who identify the methodological stance of their work. We seek to promote adoption of a framework that we consider supports the core principles of the Delphi method, to maintain research integrity.

The classic Delphi method has been described as juxtaposed between positivist and naturalistic paradigms (Hasson & Keeney, 2011). Others disagree, arguing that although analysis of results for each round may require qualitative coding or statistical summarizing (Skulmoski et al., 2007; Sobaih, Ritchie, & Jones, 2012), this process should not be perceived as transforming subjective opinion into objective data. Delphi is essentially a heuristic method, which utilizes expert opinion, experience, intuition, and tacit knowledge (Bartlett & Payne, 2013). These qualities are frequently associated with exemplary healthcare practice (Christensen & Hewitt-Taylor, 2005; Lyneham, Parkinson, & Denholm, 2008; Pretz & Folse, 2011). The research method does not result in quantitative facts, but rather the combined perspectives of experts who have knowledge of the topic and have had the opportunity to interact in ways that might be meaningful and enlightening (Cousien et al., 2014; Elkington & Lotter, 2013). The emphasis on developing understanding through the cyclical hermeneutical approach identified by Wilson (2011) through the scoping activity presents a valuable insight in the quest to identify a methodological framework for Delphi research.

### Hermeneutics

Hermeneutics is the science of interpretation (Gerber & Moyle, 2004). It is the study of understanding, to decipher meaning, and hermeneutic principles are fundamental to study for all humanistic disciplines (Palmer, 1969). The understanding or interpretation of hermeneutics itself is not without contention. Hermeneutics has been interpreted in multiple ways over time, from the theory of critical explanation of historical religious texts, to systems of interpretation. Two basic schools of thought have divided hermeneutic understanding. Hermeneutics in the tradition of Dilthey and Schleirmacher is considered as providing methodological principles for objective interpretation, whereas in the tradition of Heideggar and Gadamer hermeneutics represented ontology of relativity (Palmer, 1969). Heideggar and Gadamer uphold the view that the interpretation made by a “historian” or researcher is influenced by their pre-understandings. More simply, interpretation is necessarily subjective, as interpreter influence on the interpretation is acknowledged. Heideggar associated hermeneutics with phenomenology, and emphasized self-consciousness and the primary function of words in creating understanding, or the ontology of language. Gadamer further developed this ontological concept through his efforts in establishing hermeneutics as ontology of the event of understanding (Gadamer, 1989).

Gadamer's focus was not on processes to facilitate understanding, but rather on how understanding is shaped through the experience of exposure to text or art. Gadamer specifically noted the problematic position of healthcare in relation to the art of healing. He acknowledged the role of science, particularly in relation to medical technology and technical skill, yet argued that the art of healing comes from understanding generated through recognizing the person, not simply focusing on their body or body part. Gadamer asserted that healing is achieved through focusing on the individual and their unique situation and experience, rather than the “case” (Gadamer, 1996). The art of healthcare occurs through understanding, and similarly when understanding text and art, this occurs through engaging with a medium which causes questioning of self-understanding.

Gadamer considered understanding as a historical, dialectical, and linguistic experience. He rejected ideas of the objective subjective binary, as all human understanding is subjective (Gadamer, 1989). The concept of bracketing, as proposed by some, is discounted by Gadamer. Understanding is necessarily contextualized. Although a person may attempt to be objective, their understanding is shaped by the history of their personal knowledge and experience. Yet, new knowledge cannot emerge if the old is not challenged; therefore, an attitude of openness and interrogative communication is required. A final element of Gadamerian philosophy is that there is no final or absolute truth, when understanding is open and anticipatory. Although the Delphi research method may appear to seek consensus, it primarily seeks to facilitate the sharing of perspectives to create new shared knowledge. Therefore, we believe that the philosophical hermeneutics of Hans-Georg Gadamer may provide a suitable methodology to underpin this method.

### Delphi and Gadamer

**Figure 2:**
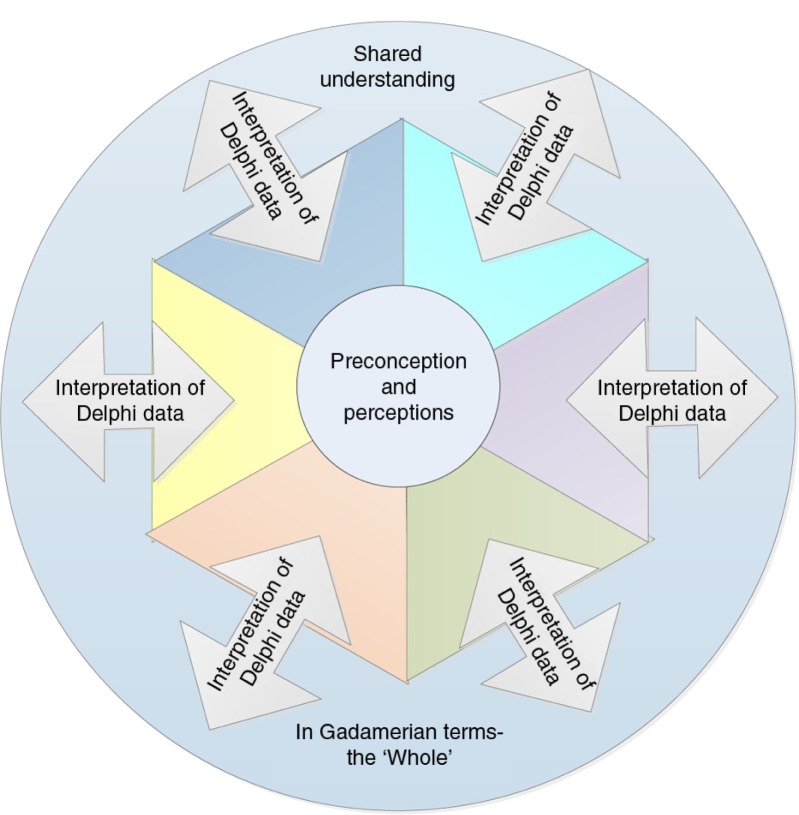
A Delphi Gadamerian Hermeneutic Circle, adapted from author.

In the health and social sciences, the emphasis of contemporary Delphi research lies in gathering expert perspectives on areas of complexity where there is a lack of common understanding, and the facilitation of reflection at each stage in the process to promote mutual understanding or highlight divergence in expert opinion. Sharing perspectives via feedback of data from each survey round enables interpretation to move from the group to individual participants, and from individual participants to the group. The iterative process involved in Delphi reflects the cyclic process of Gadamer's hermeneutic circle (Gadamer, 1989); prompting consideration of Gadamer's work in better understanding the key processes of the Delphi method. Day and Bobeva (2005) describe Delphi as an iterative feedback method that develops insight or knowledge, which is more than the sum of the parts. Through the process of Delphi, the researcher and participants create a shared understanding of the phenomena from multiple perspectives, as each survey round is informed by those which preceded it. Gadamer (1977) proposed that proper understanding is achieved through the iterative process he described as the hermeneutic circle. The hermeneutic circle explains how understanding of what Gadamer refers to as the “whole,” is constructed through repeated consideration of its components. This is expressed in Figure 2.

The hermeneutic circle depicts the process of consideration of a concept shifting between individuals and the collective, influencing the perceptions of all those involved, resulting in a shared understanding (Debesay, Nåden, & Slettebø, 2008), even when this highlights divergent views. Inherent in the process is a revision of knowledge or understanding that occurs through the process of providing feedback with each round of surveys. The role of discussion in enquiry is emphasized in Gadamerian philosophical hermeneutics (Hammersley, 2011). The process of building understanding between researcher and participants occurs in a cyclic process of interpretation, that remodels pre-existing interpretations of the phenomena being researched, co-creating meaning (Guba & Lincoln, 2005; Haverkamp & Young, 2007). The sharing of the experiences and knowledge of participants who are experts in the area of interest is a fundamental component of the Delphi method (Day & Bobeva, 2005). The development of agreement or identification of areas of disagreement, achieved through the Delphi method, demonstrates how perspectives of knowledge are shaped through interaction with others.

Researchers who work within the constructivist paradigm seek to develop an understanding of human experience through the participants’ views of the situation being studied, as reality is considered as being socially constructed and the researcher acknowledges the impact on the research of their own background and experiences (Tracy, 2012). In Gadamer's (1977) description of philosophical hermeneutics, people bring their own cultural reference point or “traditions,” from which they seek to develop an understanding of a phenomenon. Understanding and interpretation is always influenced by one's life's experiences, language, culture, and history (Frankowska & Wiechula, 2011). Delphi research is typically undertaken with experts who share similar professional backgrounds. Professional groups have their own culture, developed through professional socialization, personal experiences, and beliefs, which are founded on customary assumptions about appropriate epistemological, behavioural, and normative bases of action (Laverack, 2007). Such pre-understandings or “prejudices” shape the cognitive process of developing understanding, and are inescapable (Gadamer, 1977). Being open to other perspectives and having a willingness to reconsider the prejudices or prior assumptions on which current understandings are founded is essential (Hammersley, 2011).

**Figure 3:**
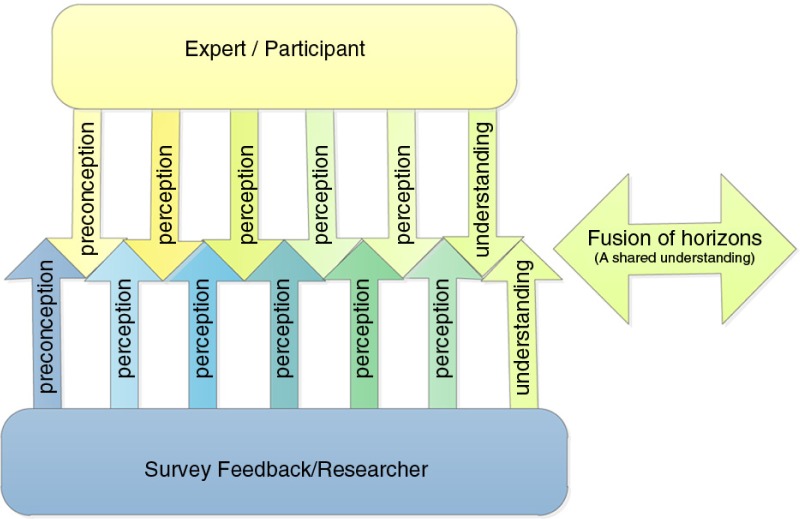
Gadamerian Fusion of Horizons, adapted from author.

Gadamerian philosophy identifies a required attitude of openness to the perspectives of others, as well as a readiness to learn and accept possible differences, referred to as *Bildung*, as personal perspectives are confronted and possibly altered by these different perspectives (Frankowska & Wiechula, 2011). This is addressed within the Delphi method through the structured reflexivity required of participants, when they consider the summary of feedback following each survey round. The reflexive dimension of understanding in Gadamerian philosophical hermeneutics distinguishes it from others, which focus on the “science of hermeneutics” (Linge, 1977, p. xii). The reflexive process acknowledges the “prejudices” and “traditions” that we bring with us, which cannot be “suspended” throughout the process of developing understanding, as understanding occurs through a process of mediation. Gadamer (1977) referred to this changing of perspectives as a “fusion of horizons,” which is illustrated in Figure 3.

Fusion occurs through the exchange of opinions, facilitating the expansion of one's personal horizon through deliberately challenging understandings and the conscious integration of the horizon of the other (Gadamer, 1977, 1989; Phillips, 2007). Further strengthening the congruency between Delphi and Gadamerian hermeneutics is the argument put forward by Linstone and Turoff (2011). They maintain that Delphi is a method for structuring a group communication process, which is not aimed to produce or force consensus, but rather to facilitate collaborative learning. Therefore awareness raising and the collective and consultative process itself is equally or possibly even more important than the outcome. The Gadamerian concept referred to as the “fusion of horizon” reflects stability of opinion in Delphi, as a new understanding is reached. Stability of opinion is consistent with the concept of data saturation used in qualitative research. When no new information is emerging from the data being collected, data saturation is considered to have occurred, signifying the natural endpoint of sampling (Liamputtong & Ezzy, 2005).

We argue that Gadamerian philosophical hermeneutics provides supportive framework consistent with the aims of Delphi, as well as elaborating a rationale for clarifying the essential components of the method. Adoption of a constructivist perspective addresses many of the criticisms and perceived limitations of this method. Most of these stem from the assumption that statistical analysis of results used in Delphi implies that it is a positivist scientific enquiry, yet the recent studies identified in the scoping review and the use of Delphi across a number of health and well-being studies indicates that it is not. The explicit limitations upon transferability of the results to other contexts, needs to be acknowledged. Examining the results of the Delphi for their cogency and plausibility is considered most appropriate and useful (McIlrath, Keeney, McKenna, & McLaughlin, 2010). Delphi is a research method that supports constructivist enquiry, suggesting that trustworthiness criteria used in qualitative research, of conformability, credibility, transferability, and dependability should replace the positivist criteria of objectivity, validity, and reliability (Day & Bobeva, 2005). The rigour of Delphi as a research method has been questioned in the past and this is likely to continue when methodological discussion is absent. Clarity is essential in the conduct and reporting of all research. Articulating methodology as well as method when reporting on research will positively influence research credibility. This may require additional time and space to present such arguments in publications, and require reviewers to be appropriately skilled in methodological assessment.

## Conclusion

We argue that methodology remains relevant and is an essential component in conducting and reporting health and well-being research. Delphi has been demonstrated to be a versatile research method used across a range of health and well-being disciplines when the goal of research is to construct a shared opinion or understanding from a group of experts of a specific phenomenon. However, Delphi was developed at a time when methodology was not recognized as foundational to research integrity. The lack of epistemological explanation for the selection of Delphi as the appropriate method for health and well-being research is illustrated by our scoping review. This omission contributes to misconceptions, resulting in unwarranted criticisms and perceived limitations of this research method. We have tried to demonstrate how Gadamerian hermeneutics may provide a suitable methodological framework for consideration when undertaking research using this method. We believe that explicitly aligning Delphi with Gadamerian philosophy clarifies the place of Delphi in health and well-being research.
